# Effect of final irrigation with sodium hypochlorite at different temperatures on postoperative pain level and antibacterial activity: a randomized controlled clinical study

**DOI:** 10.1590/1678-7757-2020-0502

**Published:** 2021-02-10

**Authors:** Ertuğrul KARATAŞ, Nilay AYAZ, Esra ULUKÖYLÜ, Mustafa Özkan BALTACI, Ahmet ADIGÜZEL

**Affiliations:** 1 Ataturk University Faculty of Dentistry Department of Endodontics Erzurum Turkey Ataturk University, Faculty of Dentistry, Department of Endodontics, Erzurum, Turkey.; 2 Ataturk University Faculty of Science, Molecular Biology and Genetics Microbiology Erzurum Turkey Ataturk University, Faculty of Science, Molecular Biology and Genetics Microbiology, Erzurum, Turkey.

**Keywords:** Sodium hypochlorite, Pain, Postoperative, Antibacterial activity

## Abstract

**Objective:**

To evaluate the effect of final irrigation of root canals with NaOCl solution at different temperatures on postoperative pain level and antimicrobial activity.

**Methodology:**

45 patients were randomly divided into three groups using a web program according to the irrigation selected: NaOCl 2ºC, NaOCl 25ºC and NaOCl 45ºC. First root canal samples were collected before treatment (S1). After chemo-mechanical preparation, final irrigation was performed with the selected irrigant (NaOCl 2ºC, NaOCl 25ºC and NaOCl 45ºC) and second samples were collected (S2). Samples were subjected to quantitative real-time polymerase chain reaction to evaluate the levels of total bacteria. The root canal treatments were completed and the participants were given instructions to record postoperative pain levels at 24, 48 and 72 hours, 5 days and 1 week after treatment using a visual analog scale (VAS).

**Results:**

The reduction in the number of total bacterial cell equivalents from S1 to S2 was statistically significant in all groups (p<0.001). The NaOCl 2˚C group reported significantly less postoperative pain than the NaOCl 45˚C group (p<0.05). Postoperative analgesic intake was significantly higher in the NaOCl 45˚C group than in the NaOCl 2˚C group (p<0.05).

**Conclusion:**

We conclude that final irrigation with NaOCl at different temperatures results in similar antibacterial effectiveness. Final irrigation with cold NaOCl (2˚C) is better than NaOCl 45˚C when comparing postoperative pain levels.

## Introduction

Sodium hypochlorite (NaOCl) is the most common irrigant used in endodontics because of its antibacterial and physicochemical properties^1^ and its unique ability to dissolve necrotic tissue remnants.^2^ Mechanical instrumentation and NaOCl irrigation have shown to dramatically reduce the bacterial load in the root canal system.^3^ Unfortunately, irrigation with NaOCl during chemo-mechanical debridement does not always result in bacteria-free root canals.^4^ Achieving bacteria-free root canals is critically important, since several studies reported that the presence of bacteria at the time of root filling significantly influences the outcome of endodontic treatment.^5-7^ Therefore, several attempts have been made to increase NaOCl antimicrobial effectiveness, such as passive ultrasonic irrigation as a supplementary disinfecting step^8^ and using NaOCl with a higher concentration,^9^ volume^10^ or temperature.^11^

Previously, Sirtes, et al. (2005) reported that NaOCl at 45˚C has better antibacterial efficacy than at 20˚C.^11^ However, preheated NaOCl superior antibacterial action and effect on postoperative pain level have not yet been clinically demonstrated; one clinical study reported that the temperature of preheated NaOCl (60˚C) starts to decrease as soon as the solution touches the root canal wall, reaching body temperature (35.7˚C) within a few minutes.^12^ Due to the ability of NaOCl to kill microorganisms in seconds,^13^ and the lack of information about the clinical antibacterial efficacy of preheated NaOCl, it is important to investigate if preheated NaOCl exerts an additional antibacterial effect and affects the postoperative pain level clinically, since the temperature of the irrigation solution is known to affect postoperative pain.^14, 15^

Consequently, there are no clinical studies to support the use of preheated NaOCl for root canal irrigation. Our randomized controlled clinical study aimed to evaluate the effect of final irrigation of root canals with NaOCl solution at different temperatures on postoperative pain level and antimicrobial activity. The null hypothesis was that the solution temperature does not change the solution antibacterial effectiveness or the postoperative pain level in patients presenting teeth with asymptomatic apical periodontitis.

## Methodology

### Case selection and treatment procedure

Forty-five patients (26 women and 19 men) presenting incisor, canine or premolar teeth with radiographic and clinical evidence of asymptomatic apical periodontitis were included in our study. This study was approved by the ethics committee of the Faculty of Dentistry, Ataturk University (Opinion No. 2017-67) and an informed consent form was signed by all participants before the treatment. The sample size (n=13) was determined using a program (GPower; Franz Faul, University of Kiel, Germany), expecting a difference of 48% in proportions of positive cultures among groups,^16^ with a 80% power and a 0.05 alpha level. It was decided to enroll 15 patients per group to increase statistical power and to account for the loss of participants during the study. Clinical registration number is TCTR20200420005.

Teeth with necrotic pulps, clinically confirmed by pulp sensibility tests and the absence of bleeding on accessing the pulp chamber, no previous history of endodontic treatment, a pocket depth of <3 mm and having a periapical lesion with a periapical index score of 3, 4 or 5 (Ørstavik, et al. (١٩٨٦)) were included.^17^ Exclusion criteria: patients that underwent treatment with antibiotics or NSAIDs within 1 month before the study; patients with any systemic disease; teeth that had received previous endodontic treatment; teeth with extensive destruction of the crown that prevented proper rubber dam isolation; and the presence of internal or external resorption. A web program (www.randomizer.org) was used to randomly assign the 45 participants into three groups (n=15). After randomization, the number of each group and of each patient were recorded. All the treatments were performed by one clinician (N.A.). Blinding the clinician to the groups was not feasible because of the recognizable temperature of the syringe. However, the patients were blinded to the groups.

Root canal samples were collected under strict aseptic technique. Before treatment, an oral rinse was performed with 0.12% chlorhexidine and before isolation of the teeth with the rubber dam, supra-gingival scaling was performed to remove plaque, followed by cleansing with pumice. Before the access cavity preparation, 30% H_2_O_2_ and 2.5% NaOCl for 30 s was used for disinfection of the crowns and surrounding structures (dam and clamp). After the disinfection protocol, the NaOCl was inactivated using 5% sodium thiosulfate. Access cavities were prepared using sterile round burs under cooling with sterile saline solution. The disinfection protocol described previously was performed again after completing access cavity preparation. To prevent the penetration of disinfectants into the pulp chamber and root canals, a sterile cotton pellet was placed on the floor of the pulp chamber. Sodium thiosulfate was used to neutralize NaOCl effectiveness and first sterility control samples were taken with sterile paper points from the coronal surface of the tooth, rubber dam, clamp and access cavity walls. Paper points were transferred to Eppendorf tubes containing a Tris-EDTA buffer (10 mmol/L Tris hydrochloride and 1 mmol/L EDTA, pH 7.6) and then the samples were kept at -80ºC until bacterial presence was assessed with quantitative polymerase chain reaction (qPCR). The teeth with negative sterility control samples for bacterial presence in the qPCR assay were included in our study.

After working length determination with an electronic apex locator (Raypex 6; VDW, Munich, Germany), the root canals were filled with sterile saline solution, not allowing them to overflow, and then a gentle filling motion was performed with a sterile #15 K-file (Mani, Tochigi, Japan). To obtain the first bacteriological sample (S1), paper points (Dentsply Maillefer, Ballaigues, Switzerland) were used to soak up the intracanal fluid in the root canal. The sampling procedure was repeated using three paper points and each paper point was left in the root canal for at least 60 seconds. Then the paper points were transferred to a tube containing a Tris-EDTA buffer. Any contact between the paper points and the cavity walls was avoided to prevent contamination during transfer of the paper points into the tubes.

Root canals were prepared using Reciproc files (R25, R40 or R50) (VDW, Munich, Germany), according to the manufacturer recommendations. During preparation, root canal irrigation was performed using 1 mL of 1% NaOCl between three pecking motions of the file and final irrigation was performed with 5 mL of 17% EDTA followed by 5 mL of 1% NaOCl (at different temperatures). According to the final irrigation, the groups were divided as follows:

NaOCl 2ºC: Syringes filled with 5 mL of 1% NaOCl were kept in a fridge at -2 ºC. Before the irrigation procedure, the syringe was removed from the fridge and when the temperature of the syringe, which was checked with a thermometer, increased to 2ºC, root canal irrigation was performed for 1 minute (the room temperature was 24.2ºC).

NaOCl 25ºC: A CanalPro syringe heater (Coltene, Altstätten, Switzerland) was used to heat a syringe filled with 5 mL of 1% NaOCl. The syringe was left in the device for 30 seconds to reach an average temperature of 26±0.2ºC, because the device cannot be set to a specific temperature. Then the syringe was removed from the device and when the temperature of the syringe, which was checked with a thermometer, decreased to 25ºC, root canal irrigation was performed for 1 minute (the room temperature was 24.2ºC).

NaOCl 45ºC: A CanalPro syringe heater (Coltene, Altstätten, Switzerland) was used to heat a syringe filled with 5 mL of 1% NaOCl. The syringe was left in the device for 20 minutes to reach an average temperature of 48±0.2ºC, because the device cannot be set to a specific temperature. Then the syringe was removed from the device and when the temperature of the syringe, which was checked with a thermometer, decreased to 45^º^C, root canal irrigation was performed for 1 minute (the room temperature was 24.2ºC).

After that, 2 mL of 0.5% sodium thiosulfate was used to inactivate the NaOCl and then the root canals were finally irrigated with distilled water. Second samples (S2) were taken as described above.

Next, paper points were used to dry the root canals and the cold lateral compaction technique was used to obturate the root canals with gutta-percha cones and sealer (Sealapex, Kerr Corporation, Orange, CA). Permanent restorations were performed using flowable and nanohybrid composite resins (3M ESPE, St Paul, MN, USA). The participants were given instructions to record postoperative pain levels at 24, 48 and 72 hours, 5 days and 1 week after treatment using an 100 mm visual analog scale (VAS) as well as recording analgesic taken on the questionnaire. If a patient was referred to an unscheduled appointment, this was also recorded.

### Quantitative real-time PCR analysis

The total bacterial counts were evaluated using 16S ribosomal RNA gene-based qPCR with QuantiTect SYBR^®^ Green PCR Kits (Qiagen) on a Rotor-Gene real-time PCR instrument (Qiagen) in a total reaction volume of 20 mL universal 16S ribosomal RNA gene-based primers (Forward primer 5’-ACTACGTGCCAGCAGCC-3’ and reverse primer 5’GGACTACCAGGGTATCTAATCC-3’); qPCR reaction conditions were 95°C for 15 min, and 40 cycles of 95°C for 15 s, 55°C for 30 s and 72°C for 30 s. For each cycle, the accumulation of PCR products was detected by monitoring the increase in fluorescence of the reporter dye (double-stranded DNA-binding SYBR Green). All measurements were performed in triplicate for samples and standards. In all experiments, triplicates of appropriate negative controls containing no DNA template were subjected to the same procedures to exclude or detect any possible contamination or carryover.

### Statistical analysis

The Poisson regression model, which is the basic approach for modeling bacterial count data, was used as previously described.^4^ For intragroup analysis, the Mann-Whitney U test was used to compare reduction of counts of total bacteria between the two samples (S1 and S2), since the data were not normally distributed. The chi-square test was used to compare the number of root canals positive for bacteria in S1 and S2 among the NaOCl 2ºC, NaOCl 25ºC and NaOCl 45ºC groups. The number of bacteria in S1 and S2 samples and the reduction (%) in the number of total bacteria from S1 to S2 among the groups were compared using the Kruskal-Wallis test. Data were analyzed with GraphPad Prism Software (GraphPad Software, San Diego, CA) at a significance level of p=0.05.

The Kruskal-Wallis test was used to compare postoperative pain values among the groups, because the data were not normally distributed. Linear regression analyses were conducted to determine the confounding effects introduced by covariates (tooth number, sex, treatment group and age). The chi-square test was used to analyze nominal data (tooth number, sex and analgesic intake). The statistical analyses for postoperative pain values were performed using IBM^®^ SPSS^®^ Statistics 20 software (IBM SPSS Inc., Chicago, IL, USA) at 5% significance level (p=0.05).

## Results

Since all the sterility control samples yielded negative results for bacteria and there was no patient loss during follow up, 45 patients (26 women and 19 men) were included in our study. Each patient contributed with 1 tooth and there was no statistically significant difference among the groups in comparison of sex, age and tooth number distribution (p>0.05) (Table 1).

**Table 1:** Distribution of patients according to age, sex, tooth number and analgesic intake

	NaOCl	NaOCl	NaOCl	p-value
	2ºC	25ºC	45ºC	
N	15	15	15	
Mean Age	28.93 ± 12.5	26.07 ± 9.83	29.13 ± 15.75	0.769
Sex				0.529
Women	9	10	7	
Men	6	5	8	
Tooth Number				0.647
#11	2	3	3	
#12	2	4	4	
#21	3	2	1	
#22	5	1	3	
#31	1	1	1	
#32	1	1	2	
#33	0	1	0	
#35	1	0	0	
#41	0	2	0	
#44	0	0	1	
Analgesic intake				0.040
No	15^a^	13^ab^	10^b^	
Yes	0^a^	2^ab^	5^b^	

Within the same row, values with the same letters were not statistically different at p=0.05

### Total bacterial counts

Table 2 shows total bacterial counts, which were analyzed using universal 16S rRNA gene-based primers, for S1 and S2 samples according to the groups. In the NaOCl 2˚C, 25˚C and 45˚C groups, a mean number of 6.47×10^6^, 5.18×10^6^and 5.76×10^6^ bacterial counts in S1 was decreased to a mean of 3.66×10^5^, 2.7×10^5^ and 3.31×10^5^ bacterial counts in S2, respectively. The reduction in the number of total bacterial cell equivalents from S1 to S2 was statistically significant in all groups (p<0.001). The percentage of reduction was 94.7, 95 and 93.9 for the NaOCl 2˚C, 25˚C and 45˚C groups, respectively. There was no statistically significant difference among the groups in terms of percentage of reduction of total bacterial counts (p>0.05).

**Table 2:** Changes in Total Bacterial Counts during Treatment

	S1	S2	Reduction (%)	p-value
**NaOCl 2°C**				
Mean	6.47 × 106	3.66 × 105	94.7	0.001
Median	6.78 × 106	3.94 × 105		
Range	1.35 × 105 – 8.21 × 106	0- 6.21 × 105		
**NaOCl 25°C**				
Mean	5.18 × 106	2.7 × 105	95.0	0.001
Median	5.55 × 106	3.01 × 105		
Range	3.22 × 106 - 6.69 × 106	0 – 5.11 × 105		
**NaOCl 45°C**				
Mean	5.76 × 106	3.31 × 105	93.9	0.001
Median	5.89 × 106	3.81 × 105		
Range	3.22 × 106 – 7.47 × 106	0 – 5.58 × 104		
p value	0.072	0.384	0.418	

S1: Sample taken before treatment

S2: Sample taken after chemo-mechanical preparation

The number of root canals positive for bacteria was also evaluated. Whereas all the S1 samples were positive for bacteria in all groups, 3 root canals from the NaOCl 2˚C group, 3 root canals from the NaOCl 25˚C group and 2 root canals from the NaOCl 45˚C group became negative for bacteria in S2 samples (Table 3). The difference among the groups in the number of root canals positive for bacteria was not statistically significant (p>0.05).

**Table 3:** The Number of Root Canals Positive for Bacteria during Treatment

	NaOCl 2°C	NaOCl 25°C	NaOCl 45°C	p-value
S1	15/15 (100)	15/15 (100)	15/15 (100)	-
S2	12/15 (80)	12/15 (80)	13/15 (86.7)	0.859

The number of cases with a positive result/ number of cases (%)

### Postoperative pain

Linear regression analyses revealed that postoperative pain level on day 1 was only influenced by the group (p<0.05). Sex, age and tooth number did not influence the postoperative pain level on day 1 (p>0.05). (Table 4).

**Table 4:** Linear Regression Model Findings for Postoperative Pain on Day 1

	B*	Standard Error	Beta	p-value
Group	17.762	6.084	0.407	0.006
Gender	-14.484	10.279	-0.201	0.167
Age	0.251	0.421	0.089	0.554
Tooth number	0.465	0.556	0.124	0.408

The mean postoperative pain level was 6.67±10.722, 16.87±27.604 and 40.0±50.709 for the NaOCl 2˚C, 25˚C and 45˚C groups, respectively. (Table 5). Statistical analysis showed that the NaOCl 2˚C group reported significantly less postoperative pain than the NaOCl 45˚C group (p<0.05). However, there was no statistically significant difference between the NaOCl 2˚C and NaOCl 25˚C groups and between the NaOCl 25˚C and NaOCl 45˚C groups in postoperative pain level on day 1 (p>0.05). Moreover, there was a statistically significant difference among the groups in postoperative analgesic intake. The number of patients that needed analgesic was 0, 2 and 5 for the NaOCl 2˚C, NaOCl 25˚C and NaOCl 45˚C groups, respectively. Postoperative analgesic intake was significantly higher in the NaOCl 45˚C group than in the NaOCl 2˚C group (p<0.05). However, there was no statistically significant difference between the NaOCl 2˚C and NaOCl 25˚C groups and between the NaOCl 25˚C and NaOCl 45˚C groups in postoperative analgesic intake (p>0.05).

**Table 5:** Postoperative pain levels according to the groups

	NaOCl	NaOCl	NaOCl	P value
	2ºC	25ºC	45ºC	
1^st^ Day	6.67^a^±10.722	16.87^ab^±27.604	40.0^b^±50.709	0,03
2^nd^ Day	8.13±10.176	14.13±27.118	25.0±35.355	0,219
3^rd^ Day	4.13±7.726	10.13±17.221	18.33±19.97	0,059
5^th^ Day	15.40±18.715	18.07±29.173	11.67±26.502	0.784
7^th^ Day	0	0	3.33±8.797	0,129

Within the same row, values with the same letters were not statistically different at P=0.05

## Discussion

Our study compared the effect of NaOCl with different temperatures on elimination of bacteria from root canals and postoperative pain level. Since there was no significant difference among the groups in antibacterial effectiveness, but the temperature of the solution affected the postoperative pain level, the null hypothesis was partially rejected. According to the results of our study, chemo-mechanical preparation and final irrigation with EDTA + NaOCl was highly effective in significantly reducing the intracanal bacterial counts, irrespective of the NaOCl temperature. This is in agreement with previous studies, which reported statistically significant reduction of bacterial counts by chemo-mechanical preparation.^4, 10, 18^ However, there was no statistically significant difference among the groups, when comparing NaOCl with different temperatures, in the removal of bacteria from root canals. Several studies investigated the antibacterial effect of NaOCl with different temperatures and conflicting results have been reported^11, 19-21^. Sirtes, et al.^11^ (2005) observed a 100-fold increase in NaOCl antibacterial efficacy when the solution was heated from 20°C to 45°C. Similarly, Giardino, et al.^20^ (2016) demonstrated better NaOCl antibacterial efficacy at 45°C than at 20°C. However, Sirtes et al. used NaOCl solution with a concentration of 0.001% and they added bacteria in phosphate-buffered saline into the NaOCl solution. That is, they did not use extracted teeth or dentine slices in their investigation to mimic clinical conditions, which may have affected the results, because the presence of organic (tissue remnants and inflammatory exudate) and inorganic matter (dentine) can weaken the antibacterial effectiveness of the NaOCl solution^22, 23^. Moreover, they incubated the bacteria in the NaOCl solution for 10 minutes. Giardino, et al.^20^ (2016) also assessed the NaOCl antibacterial efficacy after a 10-minute contact, which is a relatively longer contact time when compared with clinical conditions. Moreover, both studies were culture-based; which are less sensitive than the qPCR, which can detect as-yet-uncultivated bacteria.^24^ The methodological differences mentioned above could explain the difference in the findings of our study and the previous ones. Our findings could be explained by the strong antibacterial effect and the NaOCl concentration (1%) used in our study was efficient for killing sufficient bacteria in the root canal system. NaOCl can kill bacteria even at concentrations lower than 0.1%. Additionally, and consistent with our results, Carpio-Perochena, et al.^19^ (2015) compared NaOCl solutions with a concentration of 1% at different temperatures (22°C and 37°C) in terms of antibacterial efficacy and concluded that the temperature variation of the NaOCl is not relevant in killing or dissolving bacterial biofilms. Likewise, Gulsahi, et al.^21^ (2014) reported that there is no significant difference between the NaOCl at 25°C and 37°C in killing *Enterococcus faecalis* and *Candida albicans* for the same contact times. Therefore, it can be speculated that NaOCl with a concentration of 1% exerts a strong antibacterial efficacy that provides a substantial reduction in bacterial counts in the root canal system, regardless of the solution temperature.

Our study also evaluated the effect of the NaOCl at different temperatures on the level of postoperative pain and showed that root canal irrigation with NaOCl at 45°C resulted in a significantly higher postoperative pain value than NaOCl at 2°C. This finding is in accordance with previous studies that reported less postoperative pain with the application of intracanal cryotherapy.^14, 25^ Cryotherapy leads to reduced cellular metabolism by dropping local temperature, which causes reduced blood flow.^26^ Consequently, the effect of cryotherapy on limiting inflammation may explain the reduced postoperative pain value in NaOCl 2°C group.^27^ However, the most important finding of our study was that, although there was no statistically significant difference between the NaOCl 25°C and NaOCl 2°C groups, postoperative pain reached the highest level in the NaOCl 45°C group, with a significant difference when compared with the NaOCl 2°C group. This means that using preheated NaOCl for root canal irrigation results in a higher postoperative pain value. Additionally, in the NaOCl 45°C group, the need for analgesic intake was significantly higher than in the NaOCl 2°C group. There are no previous studies evaluating the effect of intracanal irrigation with preheated NaOCl on the level of postoperative pain, therefore, a direct comparison cannot be performed. However, the postoperative pain-enhancing effect of the preheated NaOCl can be explained by the fact that heat increases tissue temperature, which results in vasodilatation.^28^ Vasodilatation increases blood flow and allows leukocytes and plasma proteins to exit the circulation, which may cause an increase in inflammatory response, thus increasing postoperative pain.

One of the limitations of our study is that the temperature of the solution in the root canal was not constant during the irrigation procedure. De Hemptinne, et al.^12^ (2015) showed that the solution temperature (45°C) decreased to body temperature (37°C) in 60 seconds when the solution was used for root canal irrigation *in vivo*. However, since the NaOCl exerts its antibacterial efficacy in seconds,^13^ 60 seconds is enough to assess the antibacterial efficacy of the preheated NaOCl solution. Moreover, it can be claimed that a decrease in the temperature of the solution had no significant effect, since the preheated NaOCl group showed higher postoperative pain values than the NaOCl 2°C group. This means that the effect of the heat was clearly shown in our study.

## Conclusion

We conclude that preheating NaOCl does not provide any extra antibacterial effect and results in a higher postoperative pain value than the cold NaOCl when used for final irrigation of root canals of teeth with asymptomatic apical periodontitis.
